# Surgical excision and oncoplastic breast surgery in 32 patients with benign phyllodes tumors

**DOI:** 10.1186/s12957-018-1453-z

**Published:** 2018-07-24

**Authors:** Jie Ren, Liyan Jin, Bingjing Leng, Rongkuan Hu, Guoqin Jiang

**Affiliations:** 1 Department of General Surgery, The Second Affiliated Hospital of Soochow University, Suzhou, 215006 China; 2 Department of Thyroid and Breast Surgery, Traditional Chinese Medicine Hospital of Kunshan, Suzhou, 215006 China

**Keywords:** Phyllodes tumor, Core needle biopsy, Oncoplastic breast surgery, Breast reconstruction

## Abstract

**Background:**

The purpose of this study was to assess the effectiveness and safety in patients with benign phyllodes after performing local excision and following with intra-operative breast flap reconstruction.

**Methods:**

Patients (*n* = 32) with eligible breast cystosarcoma phyllodes underwent wide local excision followed by intra-operative breast flap reconstruction. Primary outcome measures included average operative time, length of in-hospital stay, postoperative recurrence, and intra-operative and postoperative complications.

**Results:**

Thirty-two patients who underwent surgical excision and oncoplastic breast surgery were evaluated using the BCCT.core software. A satisfactory symmetrical breast shape was achieved. The average operative time was 56.3 ± 8.2 min. The average postoperative duration of hospitalization was 3.7 ± 1.2 days. While there was no breast disease recurred during the 1 to 8-year follow-up period.

**Conclusions:**

Wide local excision accompanied by intra-operative breast flap reconstruction could be adopted for removing benign phyllodes tumors while retaining the basic shape of the breast.

## Background

Phyllodes tumor is a very rare breast tumor that comprises 0.3–1% of all primary breast tumors. Phyllodes tumors are often misdiagnosed as fibroadenomas, and the masses are usually so large that surgical resection may cause breast deformity.

## Patients and methods

### Patients

**Fig. 1:**
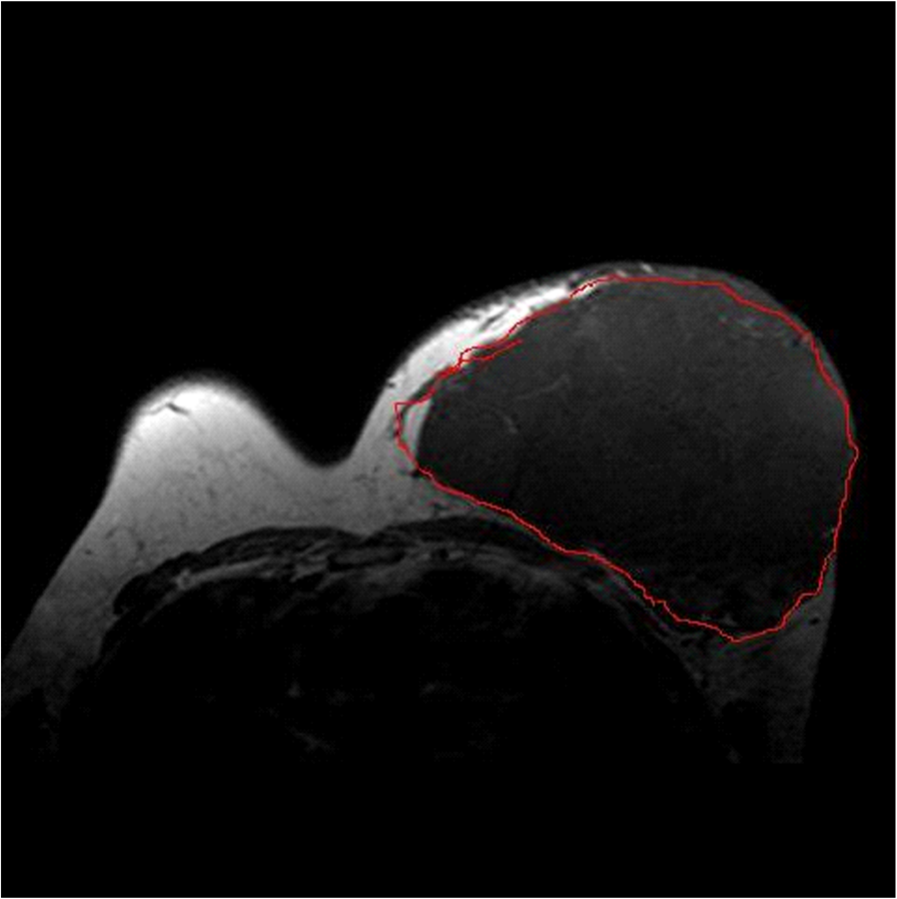
The presence or absence of breast tumor and the diameter of the tumor were determined by preoperative MRI

From January 2005 to January 2016, 32 patients who were referred to our institution (the Second Affiliated Hospital of Soochow University) for management of breast benign phyllodes tumors were retrospectively studied. All patients were well informed and signed informed consent prior to surgery. This study was approved by the ethics committee at the Second Affiliated Hospital of Soochow University. The diameter of the breast tumor was determined by preoperative ultrasonography and magnetic resonance imaging (MRI) in all patients (Fig. 1). Biopsies and pathological evaluations were performed with all patients before the surgery. Table 1 shows the baseline characteristics of the patients. Primary outcome measures included average operative time, length of in-hospital stay, postoperative recurrence, and intra-operative and postoperative complications.

**Table 1:** Baseline characteristics of the patients

Parameter	No(%)
Lump location	
Left	16 (50.0)
Right	15 (46.9)
Both	1 (3.1)
Diameter of the lump (cm)	
≤ 5	1 (3.1)
> 5,≤10	13 (40.6)
> 10,≤15	15 (46.9)
> 15	3 (9.4)
Proportion of tumor in breast (%)	
≤ 20	3 (9.4)
> 20,≤ 50	20 (62.5)
> 50	9 (28.1)
Symptoms	
Breast lump	32 (100)
Pain	3 (9.4)
Local ulceration and bleeding	1 (3.1)
Nipple retraction	0 (0)
Orange peel-like change	0 (0)
Breast operation history	
Fibroadenoma	13 (40.6)

### Methods

#### Wide local excision

We created a conventional incision around the areola, with a radial incision in the mass, forming a T-shaped incision. Areola-nipple complex dissection was performed in one patient involving an areola with skin damage. After dissecting the skin and subcutaneous tissue, the tumor was resected using a wide local dissection, removing the mass and approximately 10 mm of the surrounding tissue.

#### Breast flap reconstruction

**Fig. 2:**
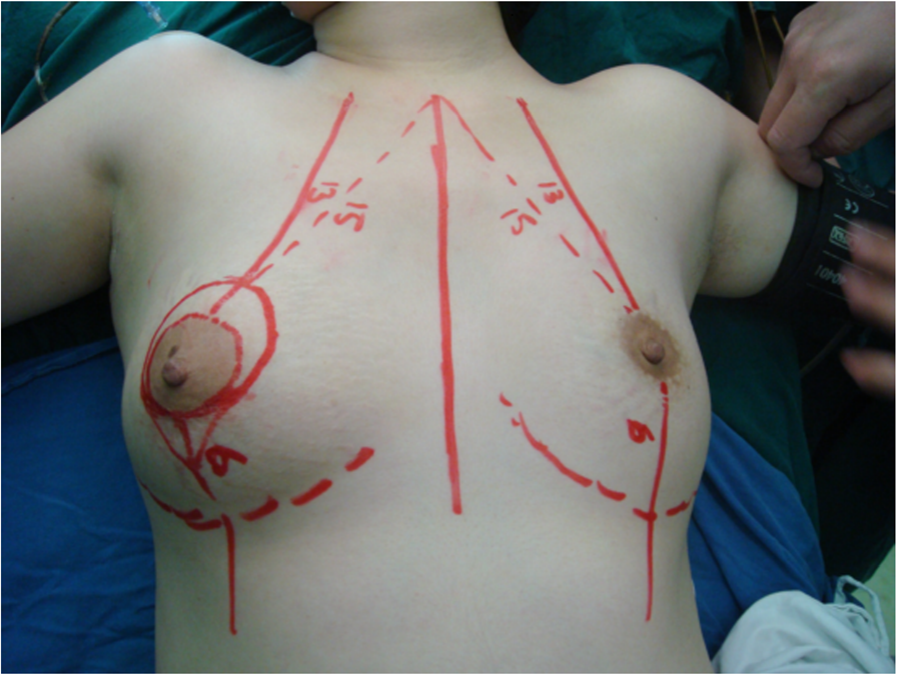
The measurement of one patient before surgery

**Fig. 3:**
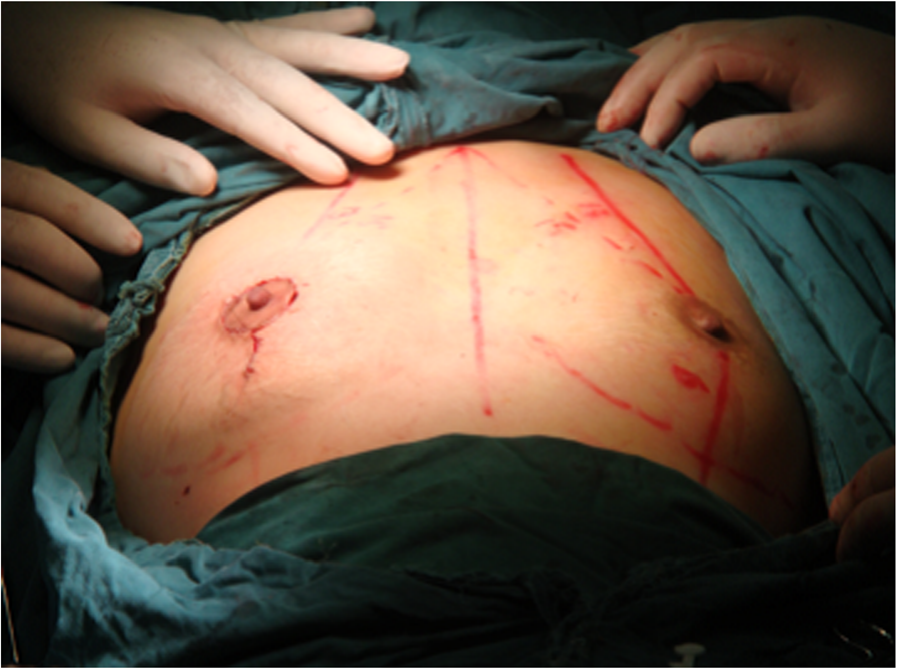
Symmetry of both sides after surgery

Before surgery, we measured the distance from the midpoint of the clavicle to the nipple and then to the lowest point of the inframammary fold (Fig. 2). The remaining breast tissue and subcutaneous fat layer were assessed after the wide local excision, and glands with more than one lobe were rotated or overlapped and then fixed to the pectoralis major fascia at the second intercostal space. We removed extra skin before suturing to ensure symmetry of both sides (Fig. 3). We sutured the incision with absorbable threads and placed a drainage tube under the incision.

#### Postoperative follow-up care

All patients received routine care and resumed oral food intake at 6 h after surgery. The drainage tube in patients was removed after the drainage volume was less than 10 ml. An elastic bandage was applied for one month.

Photographs of all 32 women, taken 1 year after surgery, were evaluated using the BCCT.core software (INSEC Porto, the University of Porto).

## Results

Wide local excision and breast flap reconstruction were completed in all patients. The average operative time was 56.3 ± 8.2 min. The average postoperative duration of hospitalization was 3.7 ± 1.2 days. All 32 cases were pathologically confirmed as benign phyllodes tumors with no margin of tumor involvement in any patient. There was no mortality, postoperative bleeding, severe breast deformity, or intra-operative complications in any of the cases (Table 2). The cosmetic outcome of the surgical excision and oncoplastic breast surgery was evaluated by BCCT.core software with 18.8% excellent, 65.6% good, and 15.6% fair. No patients experienced poor cosmetic outcomes.

**Table 2:** Surgical outcomes of the patients

	No (%)
Mean operative time (min)	56.3 ± 8.2
Postoperative stay (days)	3.7 ± 1.2
Operative complication (*n*)	
Postoperative bleeding	0
Breast severely deformed	0
Intra-operative complications	0

There was no patient experienced breast lump recurrence after a mean follow-up period of 18 months.

## Discussion

Phyllodes tumors also known as cystosarcoma phyllodes and phylloides tumor are a set of large and fast-growing masses of the breast which were first described in 1838 [1]. This rare type of breast cancer was accounted for less than 1% of all breast neoplasms and was originally considered benign; in 1931, Lee and Pack first indicated that this type of tumor also contained a malignant variant [2]. The terminology of phyllodes tumors has been adopted by the World Health Organization (WHO), which recommends that the tumors be classified as benign, malignant, or borderline according to their pathological features [3]. Though the phyllodes tumors always occur in females, but several cases were found in male patients that were diagnosed with gynecomastia [4].

As shown in this study, there were no patients that experienced postoperative bleeding, severe breast deformity, or intra-operative complications. Furthermore, we used BCCT.core to evaluate the outcome. The BCCT.core software (INSEC Porto) is an appropriate tool that is both simple and objective, and its feasibility was previously reported [5, 6]. In our opinion, surgical excision and oncoplastic breast surgery have some advantages for patients with benign phyllodes tumor.

1. The diagnosis of phyllodes tumor mainly relies on ultrasound, mammography, and MRI [7]. In mammograms, the mass usually presents as a solid, non-interrupted parenchyma with micro-calcifications. It is difficult to distinguish phyllodes tumor using MRI due to the absence of unique features. The sarcoma of phyllodes tumor is similar to undifferentiated breast cancer in intra-operative frozen sections, which often leads to a misdiagnosis of fibro-adenoma and unnecessary over-treatment. As a result, frozen sections of the breast tissue have limited value for phyllodes tumors’ diagnosis [8]. In this study, all patients underwent multi-point ultrasound-guided BARD core needle biopsy, providing sufficient tissue for routine pathological sections and immune-pathological examination.
2. Preoperative core needle biopsy of large masses may also be beneficial for developing rational surgical plans, such as local excision, wide local excision, or simple mastectomy. The biological behavior and pathological features of phyllodes tumors can vary, as histologically benign phyllodes tumors can recur and metastasize, with a recurrence rate of 10–40%. Conversely, some histologically malignant phyllodes tumors may have good clinical outcomes. In a previous study, Putti TC reported that the extent of the surgical procedure remarkably affected the rate of local recurrence [9]. Thus, the goal for the treatment of phyllodes tumors should be complete tumor removal with margins free from small lesions. Since patients in our group were preoperatively diagnosed as having benign phyllodes tumors, we used a wide (10 mm) local removal strategy. No margin of tumor involvement was pathologically detected in any patient, and no breast disease recurred during follow-up.
3. With the pursuit of increased quality of life, retention of an acceptable breast shape after the removal of large masses is a priority. In this series, even after the removal of large masses (10 mm of normal tissue), enough tissue was retained for breast reconstruction. As the breast often had a defect due to tumor resection, we made full use of the free fat layer under the entire breast and created breast tissue flaps by internal rotation or overlapping. Large breast masses can induce deformation and compression of surrounding glands as well as lead to a degree of breast displacement after lumpectomy. Thus, we outlined the breast shape, fixed remaining glands on the pectoralis major fascia at the second intercostal space, and used elastic bandages for 1 month to reconstruct a relatively intact breast shape.

Some scholars believe that benign phyllodes tumor can be cured by ultrasound-guided vacuum-assisted biopsy (UGVAB). The relapse-free survival (RFS) in patients underwent surgical excision is good than those received UGVAB alone [10]. Moreover, several previous studies mentioned that it is possible to choose an ideal prosthesis to reconstruct the tissues [11]. These can also be regarded as good methods to keep breast shape.

## Conclusions

Surgical excision and oncoplastic breast surgery are a safe and feasible procedure for patients with benign phyllodes tumors, in which rational surgical plans are developed based on preoperative multi-point ultrasound-guided BARD core needle biopsy. Utilizing wide (10 mm) local excision, making full use of the free fat layer under the entire breast, and creating breast tissue flaps can reduce recurrence and retain a good breast shape.

## Abbreviations

MRI: Magnetic resonance imaging
RFS: Relapse-free survival
UGVAB: Ultrasound-guided vacuum-assisted biopsy
